# Hashimoto's thyroiditis, vitiligo, anemia, pituitary hyperplasia, and lupus nephritis—A case report of autoimmune polyglandular syndrome type III C + D and literature review

**DOI:** 10.3389/fped.2023.1062505

**Published:** 2023-03-29

**Authors:** Yongmei Sun, Xuan Kan, Rongxiu Zheng, Liping Hao, Zongtao Mao, Ying Jia

**Affiliations:** ^1^ Department of Pediatrics, Tianjin Medical University General Hospital, Tianjin, China; ^2^ Department of Plastic and Reconstructive Surgery, The First Hospital of Jilin University, Changchun, China

**Keywords:** autoimmune polyglandular syndrome (APS) type III, Hashimoto's thyroiditis (HT), vitiligo, anemia, lupus nephritis (LN), pituitary hyperplasia

## Abstract

**Objective:**

This study aims to summarize the clinical characteristics of one teenager with autoimmune polyglandular syndrome (APS) type III C + D to improve the understanding of APS III C + D and its effect of thyroid function.

**Methods:**

This article reported the clinical manifestations, laboratory examinations, treatment methods, and outcomes of an adolescent with anemia admitted to the Pediatrics Department of Tianjin Medical University General Hospital in July 2020 and reviewed the literature.

**Results:**

A girl, aged 13 years and 1 month, was admitted to the hospital due to anemia for more than 4 years and episodic abdominal pain for 1 week. Four years ago, the girl went to a local hospital for “vitiligo”, and a routine blood test revealed anemia. The lowest hemoglobin (HGB) was 61 g/L, and the blood test revealed iron deficiency anemia. She had no menstrual cramps for 2 months. Urine routine showed protein 3+∼4+ and 258 red blood cells (RBCs)/high-power field. Urine protein was 3,380 mg/24 h. Free thyroxine was low, thyroid-stimulating hormone was >100 uIU/ml, thyroid peroxidase antibody was >1,000 IU/ml, and thyroglobulin antibody and thyrotropin receptor antibody were negative. Pituitary magnetic resonance imaging showed a mass in the sellar region with a uniform signal and a maximum height of about 15.8 mm. The result of the antinuclear antibody was 1:80 homogeneous type, and anti-dsDNA and anticardiolipin antibodies IgA and IgM were slightly higher. Thyroxine and iron were given for 1 month, menstruation resumed, and urine protein and RBC count decreased. After 5 months of treatment, free thyroid function, HGB, RBCs in urine, and pituitary returned to normal. Later, a renal biopsy showed changes in focal proliferative glomerulonephritis, and the girl was diagnosed with lupus glomerulonephritis type III. After 3 days of shock therapy with methylprednisolone, prednisone, mycophenolate mofetil, and other treatments were administrated for 1 year. At the time of writing, urine protein was 280 mg/24 h.

**Conclusion:**

Co-occurrence of Hashimoto's thyroiditis, vitiligo, anemia, pituitary hyperplasia, and lupus nephritis is rare. It is very important to pay attention to the screening of thyroid function.

## 1. Introduction

Autoimmune polyglandular syndromes (APSs) refer to the dysfunction of two or more endocrine glands under the invasion of autoimmune inflammation; it can also be a syndrome of autoimmune diseases involving non-endocrine systems. APSs are currently divided into four types (1–3), APS I, –IV. The prominent feature of APS III is the absence of adrenal involvement, which is different from APS I, II, and IV. The clinical diagnosis of APS III is defined as autoimmune thyroid disease (AITD) combined with at least one other autoimmune disease. APS III is also divided into four types (4). Among them, those with diabetes or hypophysitis are APS IIIA, those with autoimmune digestive tract diseases or pernicious anemia are APS IIIB, those with vitiligo are APS IIIC, and those with systemic lupus erythematosus (SLE) are APS IIID.

Although autoimmune thyroid disease, vitiligo, and SLE often occur in pairs, it is rare for our patient to suffer from all three of the above diseases simultaneously, involving the kidneys and pituitary gland. Thus, the clinical characteristics of this APS III C + D case were analyzed, and the related literature was reviewed to improve the understanding of APS III C + D and the effect of thyroid function.

## 2. Case description

A girl, aged 13 years and 1 month, was admitted to the hospital in July 2020 due to anemia for more than 4 years and episodic abdominal pain for 1 week. More than 4 years before admission, the girl went to a local hospital for “vitiligo”. A routine blood test revealed anemia, and the exact value of hemoglobin (HGB) was unknown. Her parents did not pay attention. She was treated with oral traditional Chinese medicine and physiotherapy to improve “vitiligo”. In the past 2 years, she has not taken any related drugs, and there is no obvious progress. Since then, routine blood monitoring showed anemia, and HGB was once 80 g/L, but no intervention was given. The girl usually had no obvious uncomfortable complaint, no skin and gum bleeding, no repeated oral ulcers, no Raynaud's phenomenon, no joint swelling and pain, and no abnormal stools and urine. There was no obvious cause for pain in her lower right abdomen and around the umbilicus 1 week before admission. Abdominal ultrasound and CT in the local hospital showed an “enlarged appendix”, and the routine blood examination showed that the HGB was as low as 65 g/L. The girl was hospitalized in the surgery department and preparing for surgery; 1 U of suspended red blood cells (+Dex) were transfused three times, Ceftriaxone was infused for 6 days, and HGB rose to 74 g/L. After her abdominal pain improved, she was referred to our department. She had no history of surgery, trauma, and poison exposure.

The girl was born at full term from a gravida 1 para 1 mother aged 37 years and had no history of birth injury or asphyxia. She had normal intellectual developmental milestones and poor academic performance. She had menarche at the age of 12 years: 4/30, regular menstruation, and low menstrual flow usually. However, she had no menstrual cramps in the past 2 months. Her father had coronary heart disease and hyperlipidemia. Her half-sister suffered from thyroid disease.

Physical examination results are as follows: pulse, 70 bpm; respiration, 17 bpm; blood pressure, 97/60 mmHg; weight, 47 kg (P50–75); height, 151 cm (P10–25). The girl had good nutritional status. Several depigmented spots of different shades and clear boundaries can be seen on the skin of the girl's face, abdomen, and feet, 0.5 cm × 0.5 cm–2 cm × 1 cm in size. It can be seen that the girl's left upper eyelid is red, swollen, and slightly prolapsed, and one small nodule with pain can be palpated on the eyelid. The palpebral conjunctiva, lips, and oral mucosa were slightly pale. The girl had no yellowing of hair, no swelling of superficial lymph nodes, less lingual papilla, cracked tongue, and no oral ulcers. The girl had no obvious enlargement of the bilateral thyroid. The girl's nail surface was of a slightly flat, rough texture. No pubic and armpit hair was seen. Other physical examinations showed no abnormality.

**Figure 1:**
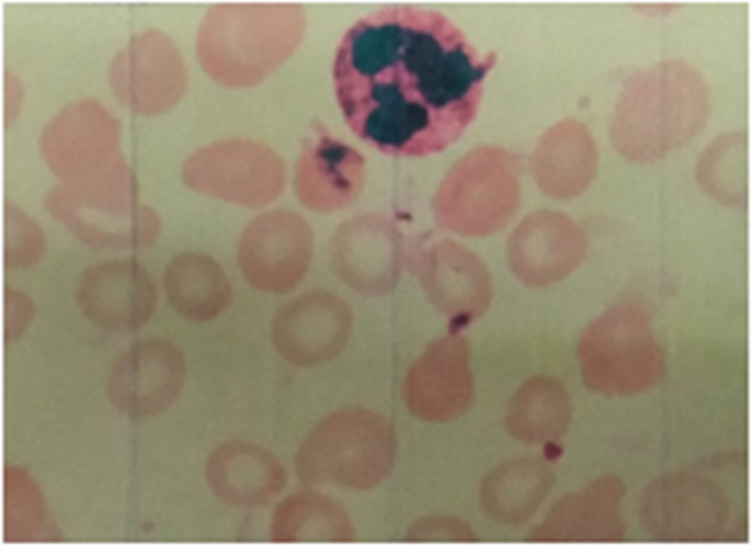
Peripheral blood smear showing small red blood cells with an enlarged central pale area.

Laboratory examination results (see Table 1) such as red blood cell (RBC), HGB, hematocrit (HCT), mean corpuscular volume (MCV), mean corpuscular hemoglobin (MCH), and mean corpuscular hemoglobin concentration (MCHC) showed lower values. Reticulocyte (RET) was slightly higher. Peripheral blood smear showed morphological small red blood cells with an enlarged central pale area (Figure 1). Ferritin (Fer) and serum iron (SI) were lower, and folic acid and vitamin B12 were normal. The above results suggested iron deficiency anemia.

**Table 1:** Laboratory examination.

Blood routine test							
RBC (4.0–4.5)*10^12^/L	HGB (110–150) g/L	HCT (37.0–43.0)%	MCV (82.0–95.0) fl	MCH (27–33) pg	MCHC (320–360) g/L	RET (0.5–1.5)%	EPO (5.4–31) mIU/ml
3.65	61	26.9	67.1	16.7	249	1.66	20.2
Ferric metabolism							
Fer (4.63- 204) ng/ml	SI (5.4–28.6) μmol/L	TIBC (40.8–76.6) μmol/L	UIBC (19.7–66.2) umol/L	Folic acid (3.10–20.5) ng/ml	Vitamin B12 (187.00–883.00) pg/ml		
<1.0	2.7	67.1	64.4	7.98	627.73		
Thyroid axis							
FT3 (2.63–5.70) pmol/L	FT4 (9.01–19.05) pmol/L	TSH (0.35–4.94) uIU/ml	TPOAb (0.00–35.00) IU/ml	TGAb (0.00–40.00) IU/ml	TRAb (0.00–1.75) IU/L		
2.89	<5.15	>100	>1,000	35.2	1.51		
Growth hormone (GH) axis							
GH (0.06–5.00) ng/ml	IGF-1 (111.00–996.00) ng/ml	IGFBP3 (2.4–10.00) μg/ml					
0.06	174	5.08					
Bone metabolism							
Ca (2.10–2.55) mmol/L	PHOS (0.80–1.45) mmol/L	ALP (40–150) U/L	25-OHD (17.5–133.00) nmol/L	PTH (1.10–7.30) pmol/L			
2.27	1.36	86	19.55	3			
Adrenocorticotropic hormone (ACTH) axis							
Blood cortisol (5–25) μg/dl	0AM	8AM	4PM	ACTH (0–46) pg/ml	0AM	8AM	4PM
	2.57	10.5	3.44		17	33.1	20.8
Gonadal axis							
FSH (follicular phase 3.03–8.08, ovulatory period 2.55–16.69, luteal phase 1.38–5.47) IU/L	LH (follicular phase 1.80–11.78, ovulatory period 7.59–89.08, luteal phase 0.56–14) IU/L	PRL (follicular phase 5.18–26.53, ovulatory period 5.18–26.53, luteal phase 5.18–26.53) ng/ml	E2 (follicular phase 21.00–251.00, ovulatory period 38.00–649.00, luteal phase 21.00–312.00) pg/ml	P (follicular phase 0.00–0.30, ovulatory period1.49–5.87, luteal phase 1.20–15.9) ng/ml	T (follicular phase 10.83–56.94, ovulatory period 10.83–56.94, luteal phase 10.83–56.94) (pg/dl)		
4.81	3.58	58.33	22	<0.10	13.07		
Electrolytes and osmolarity							
K (3.5–5.3) mmol/L	Na (135–150) mmol/L	CL (96–108) mmol/L	CO2CP (21–31) mmol/L	AG (4.00–20.00) mmol/L	Plasmatic osmolality (280–310) mOsm/kgH_2_O	Urinary osmolality (600–1,000) mOsm/kgH_2_O	
3.6	139	106	24	12.6	294	815	
Blood lipid, myocardial enzyme, and coagulation function							
TC (3.59–5.17) mmol/L	TG (0.57–1.71) mmol/L	HDL-C (0.80–2.20) mmol/L	LDL-C (1.33–3.36) mmol/L	CK (25–200) U/L	CK-MB (0–24) U/L	HDH (94–250) U/L	coagulation function (N)
4.09	1.97	0.95	2.52	40	8	202	N
Kidney function and related items							
Urea (1.7–8.3) mmol/L	CREA (44–115) mmol/L	UA (140–414) mmol/L	C-reactive protein activity (87.0–133.0)%	Free protein S content (89.3–112.5)%	Cystatin-C (0.58–1.03) mg/L	Serum β2-MG (0.80–2.90) mg/L	urine β2-MG (0.91–2.2) mg/L
2.9	44	276	106	67.2	0.74	1.74	1.85
Liver function							
TP (62–85) g/L	ALB (35–55) g/L	GLO (20–40) g/L	ALT (5–40) U/L	AST (8–40) U/L	GGT (7–49) U/L	TBIL (3.4–20.0) μmol/L	DBIL (0.1–6.8) umol/L
67	36	31	8	24	10	8.4	2
Tumor items							
CA19-9 (0.00–37.00) U/ml	CA125 (0.00–35.00) U/ml	AFP (0.00–8.78) ng/ml	CEA (0.00–5.00) ng/ml	CA15-3 (0.00–31.3) U/ml	NSE (0.00–16.30) μg/L		
49.84	35.1	1.36	1.2	6.8	15.33		
Glucose metabolism							
HbA1c (4.00–6.00)%	Fasting BG (3.60–5.80) mmol/L	Fasting insulin (4.00–18.00) mU/L	ICA (NEG)	GADA (NEG)			
5.8	5.06	7.9	NEG	NEG			
Immunologic test							
ANA < 1:80	C3 (79.00–152.00) mg/dl	C4 (16.00–38.00)mg/dl	Anti-dsDNA Ab (<18) IU/ml	ACA-IgM (<61.1) U/ml	ACA-IgA (<62.5) U/ml	ACA-IgG (<66.7) U/ml	LA test (NEG)
Homogeneous type 1:80	91.20	27.70	30.4	119.5	62.9	31.9	NEG
ASMA (NEG)	IgG (751.00–1560.00) mg/dl	IgA (82.00–453.00) mg/dl	IgM (46.00–304.00) mg/dl	IgE < 165.00 IU/ml	pANCA (NEG)	C1q-Ab (<3.18) U/ml	ASO (<116.00) IU/ml
NEG	1280	223	292	16.6	Suspiciously positive	2.8	<25.0
RF (<20) IU/ml	EAN spectrum (NEG)	CRP (<8.00) mg/L	ESR (0–20) mm/h	T/B lymphocyte subsets	IL-6 (0.1–2.9) pg/ml	Ig G subclass 4 (NEG)	
<20	NEG	<0.49	20	N	6.8	NEG	
Etiological examination							
Anti-TP (NEG)	Anti-HIV (NEG)	TB.SPOT test (NEG)	MP-IgM (NEG)	Hepatitis virus-Ab (NEG)	EBV-Ab (NEG)	CMV-IgM (NEG)	
NEG	NEG	NEG	NEG	NEG	NEG	NEG	

TIBC, total iron binding capacity; UIBC, unsaturated iron binding capacity; FT3, free triiodothyronine; TGAb, thyroglobulin antibody; TRAb, thyrotropin receptor antibody; PHOS, phosphorus; ALP, alkaline phosphatase; 25-OHD, 25-hydroxyvitamin D; E2, estradiol; P, progesterone; T, testosterone; CO_2_ CP, carbon dioxide combining power; AG, anion gap; TC, total cholesterol; TG, triglyceride; HDL-C, high-density lipoprotein cholesterol; LDL-C, low-density lipoprotein cholesterol; CK, creatin-kinase; CK-MB, creative kinase-MB isoenzyme; LDH, lactic dehydrogenase; N, normal; CREA, creatinine; UA, uric acid; β2-MG, β2-microglobulin; TP, total protein; ALB, albumin; GLO, globulin; ALT, alanine transaminase; AST, aspartate transaminase; GGT, gamma-glutamyl transferase; TBIL, total bilirubin; DBIL, direct bilirubin; AFP, alpha fetoprotein; CEA, carcinoembryonic antigen; NSE, neuron specific enolase; HbA1c, hemoglobin A1c; BG, blood glucose; ICA, islet cell antibodies; NEG, negative; GADA, glutamic acid decarboxylase antibody; C, complement; LA, lupus anticoagulant; ASMA, antismooth muscle antibody; Ig, immunogloulin; ASO, antistreptolysin "O"; RF, rheumatoid factor; ENA, extractable nuclear antigen; CRP, C-reactive protein; ESR, erythrocyte sedimentation rate; Anti-TP, Anti-treponema pallidum; HIV, human immunodeficiency virus; TB, tuberculosis; MP, mycoplasma pneumoniae; EBV, Epstein–Barr virus; CMV, cytomegalovirus.

Further examination revealed that free thyroxine (FT4) was lower than the normal value and thyroid-stimulating hormone (TSH) and thyroid peroxidase antibody (TPOAb) were significantly elevated. Thyroid ultrasound showed mild enlargement of the bilobal thyroid, thickening of the isthmus, reduced echo heterogeneity, and multiple nodules in the bilobal thyroid. Pituitary magnetic resonance imaging (MRI) showed a mass in the sellar region with a uniform signal and a maximum height of about 15.8 mm. The pituitary stalk was still in the middle, without thickening. Growth hormone (GH) was normal at a low value. Insulin-like growth factor 1 (IGF1) and insulin-like growth factor binding protein 3 (IGFBP3) were normal. The bone age was 12.7 years (0.5 years behind the patient's actual age). Prolactin (PRL) was higher. Gynecological ultrasound indicated the development of the uterus and bilateral ovaries. Blood cortisol and adrenocorticotropic hormone (ACTH) rhythm were normal. Electrolytes and plasmatic and urinary osmolarity were normal, so diabetes insipidus was not supported. Parathyroid hormone (PTH) was normal.

Rroutine urine examination showed PRO3+∼4+, BLD3+, WBC1+∼2+, RBC 258/high-power field (HPF), and WBC45/HPF. The level of 24 h urinary protein was 71.9 mg/kg.24 h, which indicated massive proteinuria. Urine protein electrophoresis showed that albumin accounted for 80%. The levels of 24 h urinary creatinine and blood and urine β2-microglobulin were normal. Urine phase-contrast microscopy indicated 95% glomerular red blood cells. Antiglomerular basement membrane (GBM) antibody was negative. Antinuclear antibody (ANA) was 1:80 homogeneous type, and perinuclear antineutrophil cytoplasmic antibody (pANCA) was suspiciously positive. Antidouble-strand DNA antibody (anti-dsDNA Ab), anti-cardiolipin antibody (ACA)-IgM, and ACA-IgA were elevated.

Triglyceride, serum carbohydrate antigen (CA) 19–9, and CA125 were slightly increased. Chest ultrasound showed that pleural effusion was seen bilaterally (12.6 mm on the left and 11.6 mm on the right). Chest CT showed ground-glass density nodules and micronodules in the lower lobe of the right lung, and the density of the cardiac chambers decreased, suggesting anemia. Abdominal ultrasound indicated that the liver, gallbladder, pancreas, spleen, kidneys, and adrenal gland were normal. Abdominal CT indicated multiple and full lymph nodes at the root of the mesentery and a small amount of pelvic effusion. Orbital CT showed enlarged left lacrimal gland and increased surrounding fat density, which was consistent with inflammatory changes.

The clinical diagnosis was Hashimoto's thyroiditis (HT) (with pituitary hyperplasia), iron deficiency anemia, glomerulopathy, hyperlipidemia, and vitiligo. The family refused renal puncture. The patient was considered to be APS IIIC.

**Figure 2:**
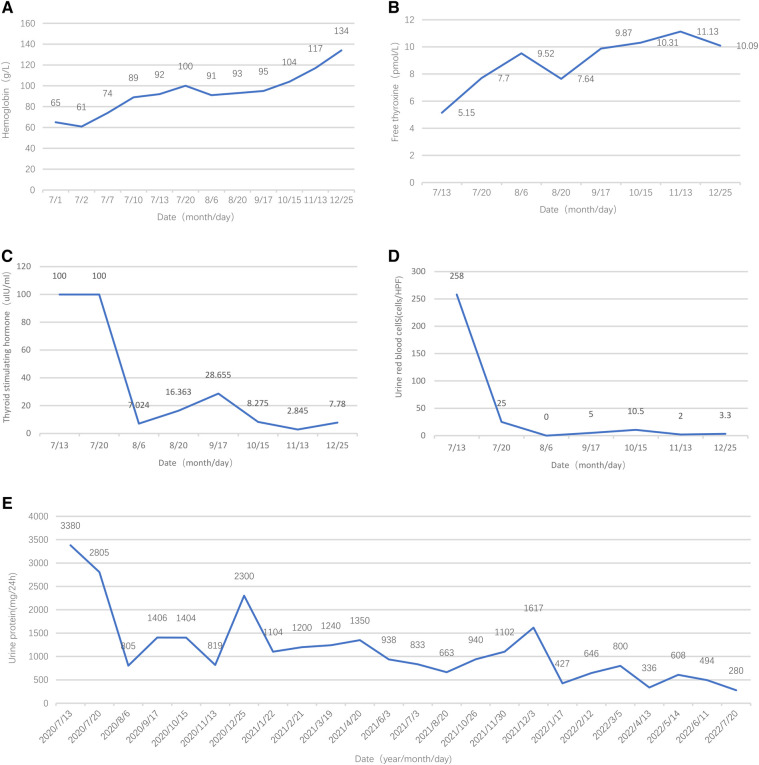
Follow-up results of hemoglobin, free thyroxine, thyroid-stimulating hormone, urine red blood cells, and urine protein.

**Figure 3:**
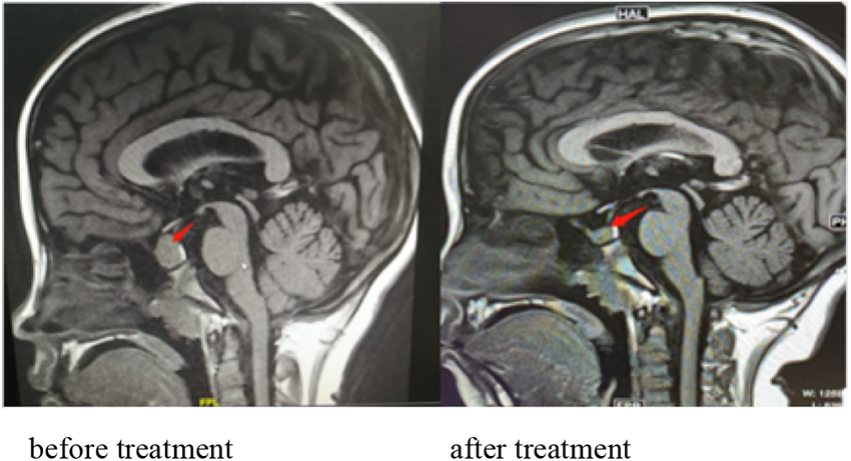
Pituitary MRI showing a mass in the sellar region with a uniform signal and a maximum height of about 15.8 mm. The pituitary stalk is still in the middle, without thickening. MRI showing that the pituitary decreased to 6.2 mm and returned to normal after treatment.

Initially, the girl was treated with levothyroxine sodium (50 μg/d) to supplement thyroxine, polysaccharide iron complex (0.15/d) to supplement iron, and captopril (12.5 mg, twice per day) to reduce urinary protein. Her condition (see Figure 2E) improved, and she was discharged from the hospital in 12 days. One month after discharge, her menstruation resumed. The hemoglobin was significantly increased, and TSH, urine protein, and red blood cells were significantly decreased (see Figures 2C–E). After 5 months of treatment, HGB and FT4 returned to the normal range (see Figures 2A,B); re-examination MRI showed that the pituitary decreased to 6.5 mm and returned to normal (Figure 3). Tumor markers returned to normal and did not support the tumor.

Until July 2021, the girl had been treated with mycophenolate mofetil (MMF) (0.75 g, twice a day) for 7 months; laboratory report showed urine occult blood ±–+, 800 mg–2,300 mg/24 h urine protein, and suspiciously positive anti-dsDNA and pANCA. Afterward, the girl was admitted to Peking Union Medical College Hospital for renal biopsy, and renal pathology showed focal proliferative glomerulonephritis (specific description: a total of 16 glomeruli can be seen in the whole film, including 1 spherical sclerosis and 1 staged sclerosis, with focal staged mesangial cell proliferation, increased mesangial matrix, and focal staged endothelial cell proliferation. A small number of capillary loops were compressed and narrowed. GBM was not significantly thickened. The subendothelial and mesangial areas were seen with erythrophilic deposition. The renal tubular epithelial cells were seen with opacity and vacuolar degeneration. There were many small focal, dense, mononuclear-dominated inflammatory cell infiltrations in the renal inerestitum). Combined with immunofluorescence, the clinical diagnosis was SLE and lupus nephritis (LN) type III. Sequential therapy include methylprednisolone 500 mg shock for 3 days and then changing to prednisone (30 mg per day). The hormone was gradually reduced to 10 mg per day for maintenance, MMF 0.75 g twice a day, tacrolimus 2 mg in the morning and 1 mg in the evening, and captopril 12.5 mg twice a day.

At the time of writing, the girl has been treated for 2 years; her height is 159.8 cm (P50), and anti-dsDNA, pANCA, and Anti-cardiolipin antibodies are all within normal ranges. The riglyceride level is normal. Urinary proteins are significantly reduced (Figure 2E).

## 3. Discussion

Autoimmune polyglandular syndrome (APS) was first reported by Schmidt in 1926 (5). APS type III is the most common type of APS in adults (3). Tian et al. (6) have well summarized the APS type III cases reported from 1989 to 2019, with a total of 64 relatively detailed cases. Our case of childhood APS type III C + D (HT, vitiligo, anemia, pituitary hyperplasia, SLE) has not been reported yet (3, 6, 7), and the specific mechanism is not fully elucidated.

### 3.1. Vitiligo and HT, SLE

A study showed that among 1,098 patients with vitiligo, nearly 20% had at least one comorbid autoimmune disease, of which 12.9% had thyroid disease and 0.3% had SLE (8). Single-nucleotide polymorphism of the tyrosine phosphatase nonreceptor 22 (PTPN22) gene is shared among patients with vitiligo and AITD (9). Studies have found that the PTPN22 1858T allele is associated with SLE and AITD (10). These findings suggest that the association observed between vitiligo, AITD, and SLE may be explained, at least in part, by the sharing of susceptibility genes.

(1) Vitiligo and HT: Vitiligo with thyroid disease is mainly vulgaris-type, and segmental-type is rare (4). This patient is consistent with literature reports (4, 11, 12).
(2) Vitiligo and SLE: The patient's condition involved multiple systems when she was admitted to the hospital; in addition to vitiligo, a disease of the immune system, there is anemia and urinary system damage, which was complex. Considering that the patient is an adolescent girl with chronic onset, we first performed related examinations for SLE. The girl also had serous cavity effusion and abnormal immunological indicators: ANA titers of 1:80, positive anti-dsDNA antibodies, and elevated anti-cardiolipin antibodies, supporting SLE. However, fever, repeated rashes, oral ulcers, photosensitivity, and joint swelling or pain were not reported. ANA titers were low, complements were not low, erythrocyte sedimentation rate was not fast, and anemia was nonhemolytic anemia; all these were insufficient evidence to diagnose SLE. Finally, 1 year later, the family members agreed to the renal biopsy, and the diagnosis of SLE and lupus glomerulonephritis type III was confirmed.

### 3.2. HT and anemia

Thyroid hormones have a wide range of physiological functions and can affect the functions of multiple organs and systems, including the hematopoietic system (13–15). The onset of hypothyroidism-related anemia is insidious, and the clinical manifestations are not specific, so it is often misdiagnosed and missed (16).

Hypothyroidism can reduce the metabolism of the hematopoietic system, reduce erythropoietin, reduce bone marrow hematopoietic function, and cause anemia. Low metabolism of iron can cause iron deficiency anemia (IDA). There may be antibodies against gastric parietal cells, resulting in atrophic gastritis and intrinsic factor deficiency. At the same time, decreased gastric acid secretion and malabsorption of iron and vitamin B12 can cause anemia. This patient has microcytic hypochromic anemia, iron deficiency, and a relative lack of erythropoietin (EPO), which is consistent with the research (15, 17). We considered that it might be related to the interference of EPO generation by hypothyroidism. However, long-term urinary occult blood can also aggravate anemia. Also, the appearance of urinary protein results in insufficient hematopoietic raw materials for hemoglobin synthesis in the body. Combined with the presence of LN in the girl, secondary kidney damage results in reduced EPO production and interference with iron metabolism, affecting bone marrow hematopoiesis.

### 3.3. HT and kidney damage

Secondary nephritis of SLE was considered according to the renal biopsy. However, through levothyroxine replacement therapy, urinary occult blood was significantly improved and urinary protein decreased, which suggested that in addition to LN, it is also necessary to pay attention to the effect of HT on the kidneys.

(1) Currently, some studies (18) believe that AITD-related nephropathy may be caused by the deposition of thyroid peroxidase and thyroglobulin outside the glomerular basement membrane, resulting in the formation of *in situ* immune complexes or the formation of circulating immune complexes with antibodies in the glomerulus. In hypothyroidism, the glomerular filtration barrier is damaged, the glomerular capillary basement membrane is thickened, the permeability is enhanced, a large amount of urine protein is lost, and the plasma colloid osmotic pressure is reduced, further aggravating the edema. In addition, impaired immune tolerance to megalin, a thyroid-stimulating hormone-regulated glycoprotein expressed on thyroid cells, also contributes to its pathogenesis. Serum total thyroxine (TT4) and FT4 were negatively correlated with massive proteinuria.

At the same time, chronic kidney disease can also lead to changes in thyroid hormones and thyroid function (19–21). Patients with massive proteinuria have high levels of TSH, which is related to the loss of thyroid hormones and thyroid-binding globulin from the urine (21). Also, the conversion of T4 into triiodothyronine (T3) decreases, thyroid-binding globulin decreases, and blood T3 and T4 decrease. Correspondingly kidney damage can exacerbate Hashimoto's thyroiditis.

(2) HT and SLE: The coexistence of the two kinds of diseases may be related to both having the susceptibility gene 5q14.3–q15 (22) and the high expression of HLA-B8 and DR3 (23). Th1 predominance is also an immunological mechanism for the coexistence of SLE and AITD (24). The thyroid itself is also a part of the systemic organ damage in SLE, which may cause thyroid dysfunction. In terms of the severe complications of SLE, those with thyroid diseases carried higher risks for lupus nephritis involvement (25). There is a correlation between hypothyroidism and lupus activity (26). Also, decreased albumin and increased serum creatinine are associated with hypothyroidism (25–29). However, antithyroid antibodies (ATAs) were not associated with SLE activity. The positive rate of ATA and the incidence of abnormal thyroid function in children with LN were higher than those in the general population. When SLE is severely active, it affects the regulation of the hypothalamus -pituitary -thyroid (HPT) axis, and the level of T3 decreases, which is proportional to the severity and duration of the disease (30).

### 3.4. HT and pituitary hyperplasia, growth impairment, and disorder of reproductive function

#### 3.4.1. HT and pituitary hyperplasia

Pituitary hyperplasia can be secondary to primary hypothyroidism (PPH) (31–33), and its degree correlates with the severity of hypothyroidism (34).

Pituitary tumor-like hyperplasia is due to primary hypothyroidism feedback activation of the HPT axis, resulting in increased thyrotropin-releasing hormone (TRH), stimulation of anterior pituitary TSH cell proliferation, pituitary enlargement (34), and even adenomas. Due to an insufficient understanding of primary hypothyroidism, there have been many clinical reports of surgical treatment of pituitary tumors. Correct and timely diagnosis can avoid unnecessary surgery or inappropriate drug treatment. Through thyroid hormone replacement therapy, with the recovery of thyroid function, the secondary pituitary hyperplasia or adenoma will gradually shrink until it disappears. The patient in this case was a girl with hyperplasia of the pituitary gland. After 5 and a half months of treatment, the MRI scan showed that the pituitary was significantly reduced to normal (Figure 3).

#### 3.4.2. HT and growth impairment

The thyroid hormone mediates bone maturation and development of the skeleton *via* its direct and permissive effects on GH (35).

On the one hand, *via* specific membrane transporters, T3 enters the target cell nucleus where it binds and activates either thyroid hormone receptor α or β (TRα, TRβ). TRα is the main receptor expressed in the skeleton and mediates T3 action in bone and cartilage. TRβ mediates negative feedback control of the HPT axis (36, 37). Thyroid hormone mediates the growth, development, and maturation of the skeleton by regulating chondrocyte proliferation, promoting differentiation of bone progenitor cells, mineralization, and angiogenesis.

In juvenile hypothyroidism, skeletal maturation is predominantly affected by delayed fusion of the epiphysis and delayed bone age. It leads to delayed skeletal development, linear growth retardation, and short stature.

On the other hand, the thyroid hormone also has a permissive role in the action of GH by promoting GH secretion from the pituitary, as well as GH-dependent IGF 1 production in the bone (35). GH secretion decreases when thyroxine level decreases, eventually leading to impaired height.

Prompt treatment of children with thyroid hormone replacement induces a period of fast growth in which skeletal maturation and bone age are also accelerated (36). However, whether the predicted adult height is attainable depends on the severity of hypothyroidism and its duration before thyroid hormone replacement begins. The girl reported in the present case had a deceleration of growth, delayed bone age (0.5 years behind the patient's actual age) as shown by imaging examination, a lower level of GH than the normal range, and a height increase of 8.8 cm after 2 years of thyroid hormone treatment, with an increase in height percentile from P10–25 to P50.

#### 3.4.3. HT and disorder of reproductive function

HT can lead to a disorder of reproductive function through direct and indirect interactions with the hypothalamus–pituitary–ovarian axis and the reproductive organs (38). First, the synergistic effect between follicle-stimulating hormone (FSH) and T3 can directly stimulate the function of granulosa cells and the formation of luteinizing hormone (LH)/human chorionic gonadotropin receptors (38, 39). Thyroid receptors exist on oocytes, and thyroid antibodies exist in follicular fluid (38, 40–42).

Severe juvenile hypothyroidism can result in follicle dysplasia, ovulatory dysfunction, and insufficient corpus luteum development with low progesterone production. It also affects the function of the ovaries and leads to menstrual disorders and delayed sexual maturation.

Due to primary hypothyroidism feedback activation of the HPT axis, TRH increased. TRH leads to the proliferation of prolactin cells and the increase of PRL (43). Hyperprolactinemia is also a common cause of ovulatory dysfunction (44). It may impair the pulsatile secretion of gonadotropin-releasing hormone (GnRH) and result in ovarian dysplasia.

In overt thyroid dysfunction, rapid initiation of thyroid hormone therapy can make endocrine hormones return to normal. Also, menstruation can be restored (32, 43) and sexual development can be normalized. The girl in this case had stopped menstruation for 2 months, and prolactin was slightly higher. After 2 months of treatment, menstrual cramps began again and prolactin had returned to normal, which is consistent with reports.

### 3.5. Tips

This case suggests that (1) when there is multisystem immune damage, the possibility of thyroid involvement, APS, and SLE should be considered if it cannot be explained by common etiologies or if it cannot be cured for a long time. (2) When chronic anemia is inconsistent with renal function, or there is unexplained proteinuria or occult blood in urine, please pay attention to screening for hypothyroidism to reduce unnecessary blood transfusions. (3) The diseases in children with no obvious symptoms of abnormal thyroid function and mild thyroid enlargement are difficult to diagnose and can be misdiagnosed and mistreated easily. Thus, regardless of whether the thyroid is enlarged or not, in the presence of immune system diseases or the involvement of multiple systems, it is very important to screen free thyroid function and antibodies. (4) For pituitary hyperplasia, we should be wary of severe hypothyroidism and check free thyroid function in time to reduce unnecessary surgery.

In conclusion, the clinical manifestations of APS are complex and diverse. Patients diagnosed with APS should be followed up regularly to be alert to other comorbidities. Also, this patient should be alert to the occurrence of polyphospholipid syndrome in the follow-up process in the future. The pathogenic mechanism of APS type III C + D in children is still fully unclear, and the long-term effect of the therapy still needs further observation in larger sample sizes over longer time periods.

## Data Availability

The original contributions presented in the study are included in the article/Supplementary Material; further inquiries can be directed to the corresponding author.
